# Transcriptome Profiling Reveals Novel Candidate Genes Related to Hippocampal Dysfunction in SREBP-1c Knockout Mice

**DOI:** 10.3390/ijms21114131

**Published:** 2020-06-10

**Authors:** Mary Jasmin Ang, Juhwan Kim, Sueun Lee, Sung-Ho Kim, Jong-Choon Kim, Tae-Il Jeon, Seung-Soon Im, Changjong Moon

**Affiliations:** 1 Department of Veterinary Anatomy, College of Veterinary Medicine and BK21 Plus Project Team, Chonnam National University, Gwangju 61186, Korea; 166371@jnu.ac.kr (M.J.A.); juhwankim9@gmail.com (J.K.); leese8892@naver.com (S.L.); shokim@chonnam.ac.kr (S.-H.K.); toxkim@chonnam.ac.kr (J.-C.K.); 2 Herbal Medicine Resources Research Center, Korea Institute of Oriental Medicine, 111 Geonjae-ro, Naju-si 58245, Korea; 3 Department of Animal Science, College of Agriculture and Life Science, Chonnam National University, Gwangju 61186, Korea; tjeon@chonnam.ac.kr; 4 Department of Physiology, Keimyung University School of Medicine, Daegu 42601, Korea; ssim73@kmu.ac.kr

**Keywords:** SREBP-1c, RNA-sequencing, hippocampus, lipid metabolism, brain, mouse model, schizophrenia, transcriptome profiling, novel genes

## Abstract

Lipid homeostasis is an important component of brain function, and its disturbance causes several neurological disorders, such as Huntington’s, Alzheimer’s, and Parkinson’s diseases as well as mood disorders. Sterol regulatory element-binding protein-1c (SREBP-1c) is a key modulatory molecule involved in lipid homeostasis in the central nervous system. However, little is known about the biological effects of SREBP-1c in the brain. Our previous study uncovered that mice deficient in SREBP-1c exhibit schizophrenia-like behaviors. To investigate whether there are novel molecular mechanisms involved in the neurological aberrations caused by SREBP-1c deficiency, we analyzed the transcriptomes of the hippocampus of SREBP-1c knockout (KO) mice and wild-type mice. We found seven differentially expressed genes (three up-regulated and four down-regulated genes) in the hippocampus of SREBP-1c KO mice. For further verification, we selected the three most significantly changed genes: glucagon-like peptide 2 receptors (GLP2R) involved in hippocampal neurogenesis and neuroplasticity as well as in cognitive impairments; necdin (NDN) which is related to neuronal death and neurodevelopmental disorders; and Erb-B2 receptor tyrosine kinase 4 (ERBB4) which is a receptor for schizophrenia-linked protein, neuregulin-1. The protein levels of GLP2R and NDN were considerably decreased, but the level of ERBB4 was significantly increased in the hippocampus of SREBP-1c KO mice. However, further confirmation is warranted to establish the translatability of these findings from this rodent model into human patients. We suggest that these data provide novel molecular evidence for the modulatory role of SREBP-1c in the mouse hippocampus.

## 1. Introduction

Sterol regulatory element-binding proteins (SREBPs) are a family of transcription factors that regulate lipid homeostasis by controlling the synthesis of cholesterol, fatty acids (FA), triacylglycerol, and phospholipids [1]. The SREBP family includes three isoforms: SREBP-1a, SREBP-1c, and SREBP-2 [2]. SREBP-2 is encoded by the sterol regulatory element-binding transcription factor 2 (*Srebf2*) gene while both SREBP-1a and -1c are encoded by the *Srebf1* gene as it has independent promoters that utilize alternate first exons that are then spliced into common exons [1,3]. SREBP-1c primarily regulates FA and triglyceride synthesis, SREBP-2 controls cholesterol synthesis, and SREBP-1a participants in the roles of both SREBP-1c and SREBP-2 [4]. When the cellular lipid level is low, the SREBP cleavage activating protein transfers SREBP precursors from the endoplasmic reticulum to the Golgi apparatus, in which two distinct proteases sequentially cleave the SREBP precursors, which results in the release of the active forms [5,6]. The active forms subsequently translocate to the nucleus and regulate the transcription of target genes by binding to enhancer (E)-boxes and sterol regulatory element deoxyribose nucleic acid (DNA) sequences [7].

As SREBPs primarily play a role in lipid homeostasis by controlling the production of fatty acids and cholesterol, they have been widely studied in the liver and adipose tissue [8]. Lipid homeostasis plays an important role in neuronal function especially in the regulation of the membrane’s function between the intracellular and extracellular spaces, modulation of synaptic outputs, and release of lipid-derived second messengers [8,9]. Accumulating evidence has revealed that a disturbance in lipid homeostasis can cause several brain diseases, such as Huntington’s disease [10], Alzheimer’s disease [11], Parkinson’s disease [12], and mood disorders [9]. This confirms the importance of adequate SREBP function in the brain. Among the SREBP family isoforms, SREBP-1c is the predominant isoform that is considered to play a central role in lipid homeostasis [13,14]. Recently, we found that the deficiency of SREBP-1c induces schizophrenia-like behavior in mice [15], and further explore the mechanisms involved in these behavioral aberrations, we sought to identify differentially expressed genes (DEGs) in the hippocampus of SREBP-1c knockout (KO) mice compared to in that of wild type (WT) littermates.

In this study, we aimed to identify the transcript level change in the hippocampus of SREBP-1c KO mice. We examined DEGs that were identified by performing an enrichment analysis. Additionally, a quantitative real-time polymerase chain reaction (qRT-PCR), western blot analyses, and immunohistochemical analyses were performed to verify the transcript and protein level changes.

Our main conclusions outline that the presence of alterations in the expression of novel gene candidates in the hippocampus of SREBP-1c KO mice is closely related to the development of schizophrenia-like behavior; further, we have identified the differential gene expression of various genes, which are potentially responsible for changes in signaling transduction, cell differentiation, and cell survival within the hippocampal cell population.

## 2. Results

### 2.1. Validation of the Expressions of the SREBP Isoforms in the Mouse Hippocampus

**Figure 1:**
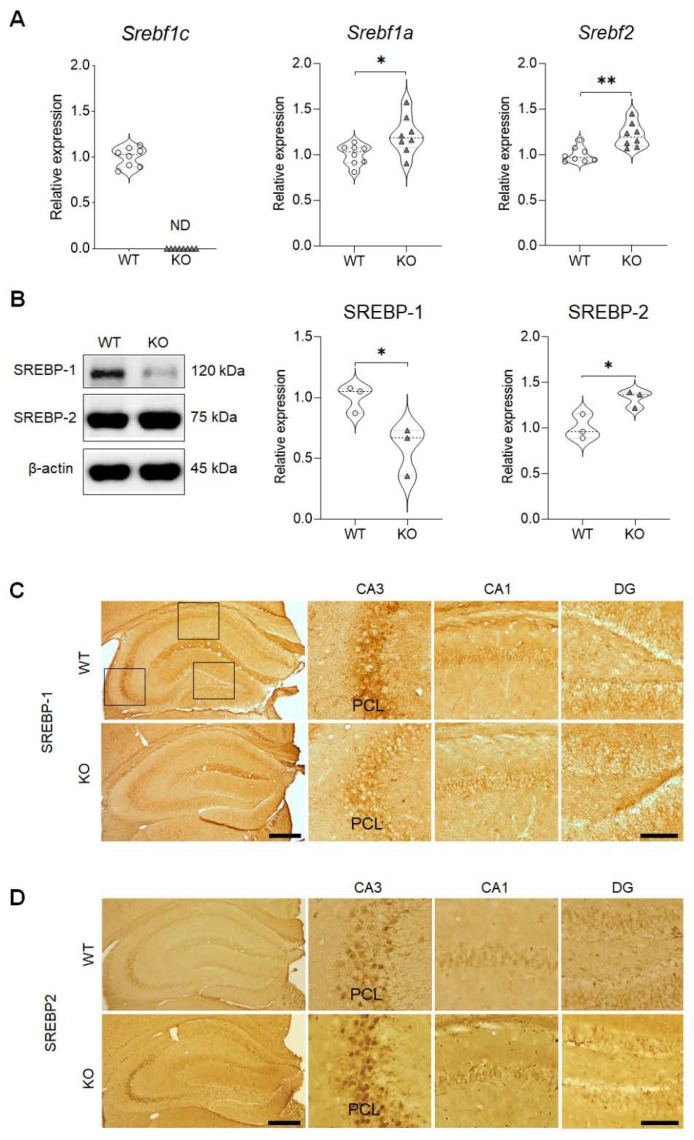
mRNA and protein expressions of the SREBP isoforms in the hippocampus of SREBP-1c KO mice. (**A**) qRT-PCR results confirm the *Srebf1c* (transcript variant 3, left panel), *Srebf1a* (transcript variant 1, middle panel), and *Srebf2* (right panel) transcript levels (n = 8, * *p* < 0.05 and ** *p* < 0.01 compared to WT mice). (**B**) Western blot analyses reveal that SREBP-1 expression is significantly decreased in contrast to SREBP-2 expression that is slightly increased in the hippocampus (n = 3, * *p* < 0.05 compared to WT mice). (**C**,**D**) Immunohistochemical analyses reveal that both SREBP-1 (**C**) and SREBP-2 (**D**) are expressed in the CA1, CA3, and DG subregions of the hippocampus (with especially intense immunoreactivities in the PCL of the CA3 subregion). Scale bars = 100 µm (left panels), 50 µm (Insets). CA, cornu ammonis; DG, dentate gyrus; KO, SREBP-1c knockout mice; mRNA, messenger ribonucleic acid; ND, not detected; PCL, pyramidal cell layer; qRT-PCR, quantitative real-time polymerase chain reaction; *Srebf*, sterol regulatory element-binding transcription factor; SREBP, sterol regulatory element-binding protein; WT, wild-type mice.

The messenger ribonucleic acid (mRNA) expressions of the *Srebf1* isoforms and of *Srebf2* were examined in the hippocampus (n = 8 mice/group) of the WT and SREBP-1c KO mice. *Srebf1c* mRNA was not detected in the SREBP-1c KO hippocampi (Figure 1A, left panel). However, the mRNA levels of *Srebf1a* (mean (M) = 1.214, standard deviation (SD) = 0.2068; t(14) = 2.587, *p* = 0.0215; Figure 1A, middle panel) and *Srebf2* (M = 1.214, SD = 0.1326; t(14) = 3.857, *p* = 0.0017; Figure 1A, right panel) were slightly but significantly higher in the SREBP-1c KO hippocampi compared to in the WT hippocampi.

To determine if the protein expression pattern that we uncovered corresponded with the results of the qRT-PCR analyses, western blot analyses were performed for SREBP-1 and SREBP-2 (Figure 1B). As expected, in the KO hippocampus, the protein levels associated with SREBP-1 were significantly decreased by ~42% (M = 0.5846, SD = 0.2010; t(4) = 3.129, *p* = 0.0352), but SREBP-2 expression was slightly but significantly increased (M = 1.327, SD = 0.09317; t(4) = 3.435, *p* = 0.0264). These results validated the results of both the RNA-sequencing (RNA-seq) analysis and qRT-PCR.

Additionally, these findings were confirmed by the immunohistochemical results that revealed a similar decrease in the expression pattern of SREBP-1 (Figure 1C) and a slight increase in the expression of SREBP-2 (Figure 1D) in the SREBP-1c KO hippocampi. SREBP-1 and SREBP-2 expressions were observed in all the subregions of the hippocampus, including in the cornu ammonis 1 (CA1), cornu ammonis 3 (CA3), and dentate gyrus (DG). Particularly intense immunoreactivities for both SREBP-1 and SREBP-2 were localized in the pyramidal cell layer (PCL) of the hippocampal CA3 subregion. Following the validation of the ablation of the *Srebf1c* gene in the KO mice, we conducted the RNA-seq procedure.

### 2.2. Identification of DEGs in the Hippocampus of SREBP-1c KO Mice and Protein–Protein Interaction (PPI) Analysis

**Figure 2:**
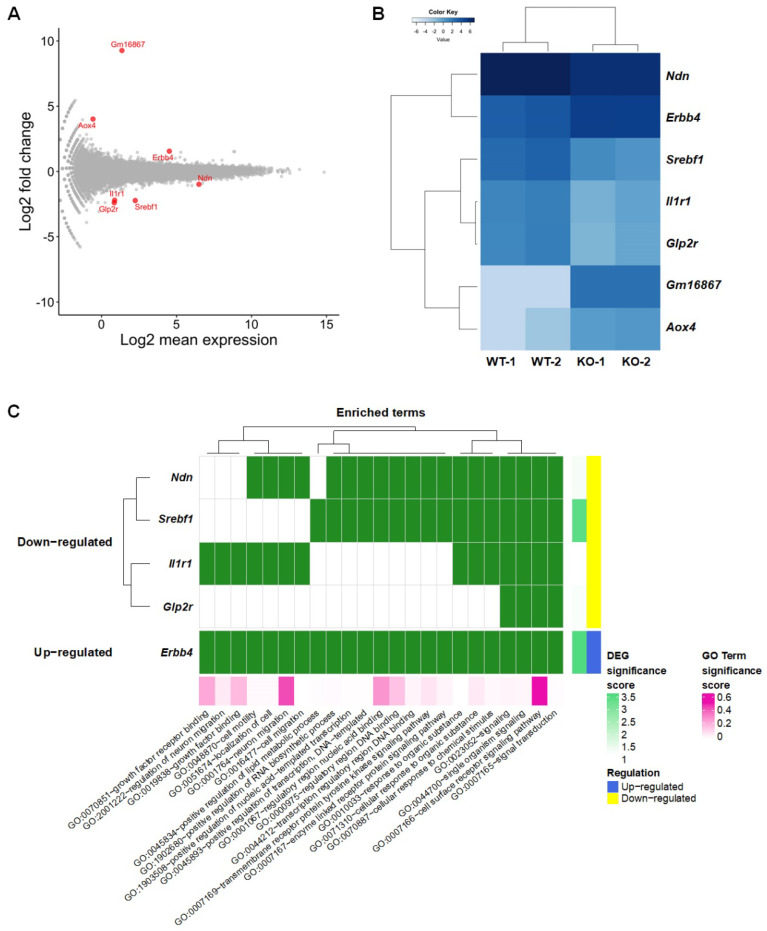
Distinct gene expression changes in the hippocampus of SREBP-1c KO mice. (**A**) MA plot and (**B**) Heat map showing DEGs (FDR < 0.05, |Log2 fold change| ≥1). (**C**) Gene ontology enrichment analysis. DEG significance score is expressed as −log2 (FDR). *Aox4*, aldehyde oxidase 4; DEG, differentially expressed genes; *Erbb4*, Erb-B2 receptor tyrosine kinase 4; FDR, false discovery rate; *Glp2r*, glucagon-like peptide 2 receptor; *Il1r1*, interleukin 1 receptor, type I; *Ndn*, necdin; *Srebf1*, sterol regulatory element-binding transcription factor 1.

To identify DEGs in the SREBP-1c KO mice in comparison to in the WT mice, the EdgeR package was chosen since, up to 2018, it has been the most widely used by researchers [16] and has demonstrated better performance in identifying true positives [17] compared to Cuffdiff2 and DESeq. The ExactTest function in the EdgeR package was used to analyze DEGs [18]. We detected seven genes by using the false discovery rate (FDR) < 0.05 criteria in an RNA-seq dataset (Figure 2A and Supplementary Table S1). The divergence between the gene expression of the WT and SREBP-1c KO mice was confirmed by hierarchical clustering (Figure 2B). The results of a gene ontology analysis using the list of seven DEGs suggest that the signaling pathways, cell/neuron migrations, cellular responses, transcription, cell motility, and localization of cells are affected in SREBP-1c KO mice (Figure 2C and Supplementary Table S2) because the *Srebf* gene is suggested to act as a transcription factor and signal transduction regulator that controls lipid metabolism and insulin signaling [2].

Additionally, using the Search Tool for the Retrieval of Interacting Genes (STRING) database, among the DEGs identified only Erb-B2 receptor tyrosine kinase 4 (*Erbb4*) and interleukin 1 receptor type I (*Il1r1*) were found to interact with *Srebf*s [19]. *Erbb4* had predicted interactions with *Srebf1* predicted by co-citation analysis with pre-existing scientific texts and gene fusion analysis. For example, a previous study implicated *Erbb4* as an activator of *Srebf2* mediating increases in low-density lipoprotein uptake and cholesterol biosynthesis in mammary epithelial cells [5]. *Il1r1*, on the other hand, had predicted interactions with *Srebf1* evidenced by text-mining, gene fusion analysis, gene co-expression, and a known interaction based on curated interaction records (Supplementary Figure S1).

### 2.3. Validation of the DEGs in the Hippocampus of SREBP-1c KO Mice

**Figure 3:**
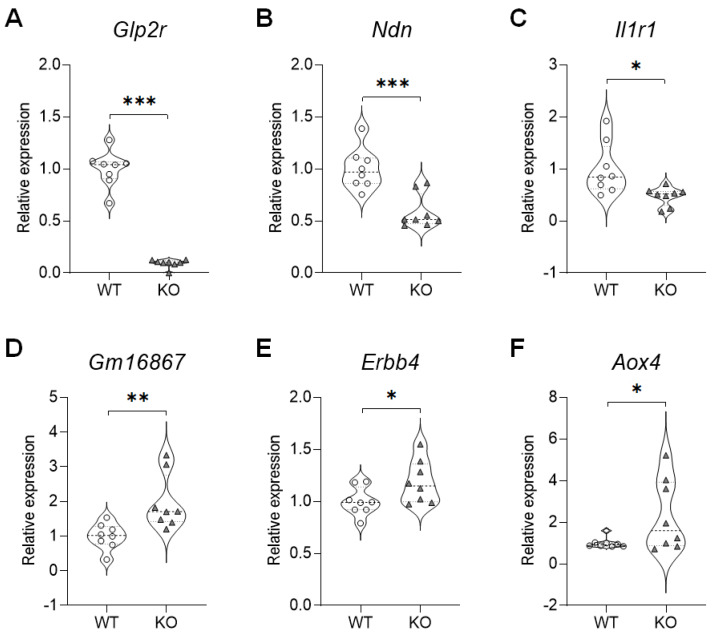
qRT-PCR validation of selected DEGs in the hippocampus of SREBP-1c KO mice. (**A**) *Gip2r*, (**B**) *Ndn*, (**C**) *Il1r1*, (**D**) *Gm16867*, (**E**) *Erbb4*, (**F**) *Aox4* (n = 8, * *p* < 0.05, ** *p* < 0.01 and *** *p* < 0.001 compared to WT mice). *Aox4*, aldehyde oxidase 4; DEG, differentially expressed genes; *Erbb4*, Erb-B2 receptor tyrosine kinase 4; *Glp2r*, glucagon-like peptide 2 receptor; *Il1r1*, interleukin 1 receptor type I; KO, SREBP-1c knockout mice; *Ndn*, necdin; qRT-PCR, quantitative real-time polymerase chain reaction; WT, wild-type mice.

To determine if the gene expressions detected via the qRT-PCR were similar to those detected via the RNA-seq analyses, we analyzed selected genes based on gene expression and biological relevance (Figure 3). A gene expression validation technique revealed that in the hippocampus of the SREBP-1c KO mice, the expressions of the glucagon-like peptide 2 receptor (*Glp2r*) (M = 0.09292, SD = 0.03947; t(14) = 14.38, *p* < 0.0001), necdin (*Ndn*) (M = 0.5874, SD = 0.1639; t(14) = 4.559, *p* = 0.0004), and *Il1r1* (M = 0.4722, SD = 0.1774; t(14) = 2.835, *p* = 0.0132) genes significantly decreased (Figure 3A–C), while the levels of *Gm16867* (M = 1.965, SD = 0.7913; t(14) = 3.124, *p* = 0.0075), *Erbb4* (M = 1.188, SD = 0.2059; t(14) = 2.159, *p* = 0.0487), and aldehyde oxidase 4 (*Aox4*) (M = 2.323, SD = 1.724; t(14) = 2.148, *p* = 0.0497) were significantly increased (Figure 3D–F).

**Figure 4:**
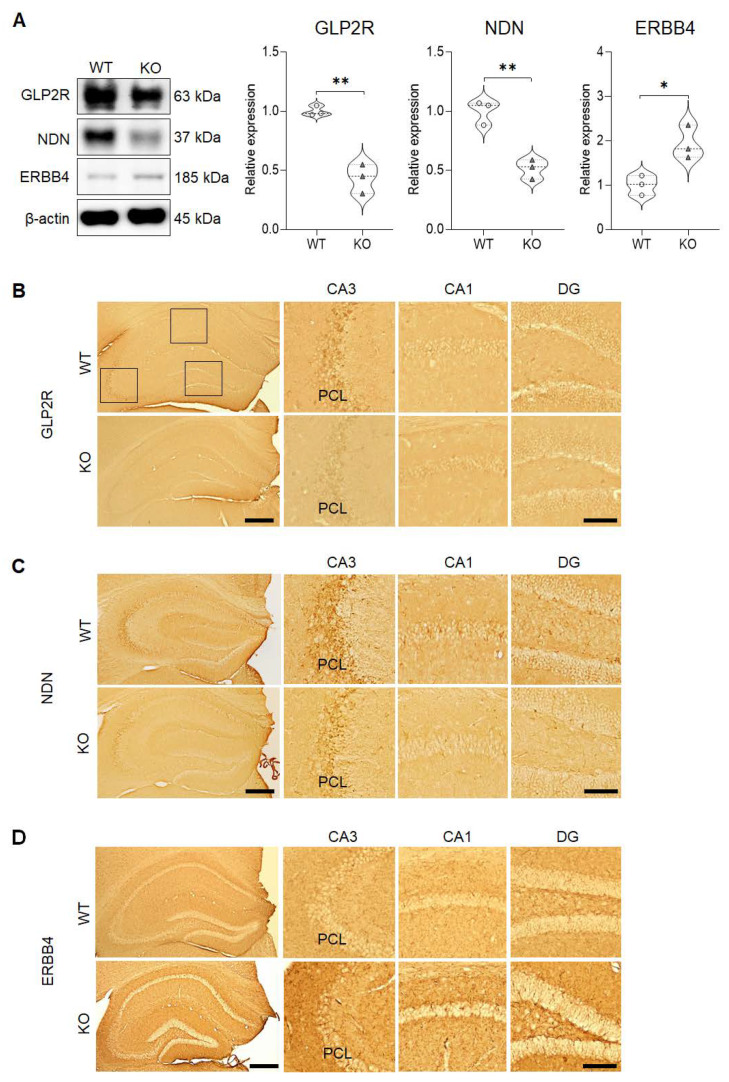
Validation of GLP2R, NDN, and ERBB4 protein expressions in the hippocampus. (**A**) Western blot analyses confirm that both GLP2R and NDN expressions are significantly decreased, while ERBB4 expression is significantly increased in the hippocampus of SREBP-1c KO mice compared to in those of WT mice (n = 3, * *p* < 0.05 and ** *p* < 0.01 compared to WT mice). (**B**–**D**) Immunohistochemical analyses reveal that both GLP2R (**B**) and NDN (**C**) are expressed in the CA1, CA3, and DG subregions of the hippocampus (with especially intense immunoreactivities in the PCL of the CA3 subregion), while ERBB4 (**D**) is expressed extensively in all subregions of the hippocampus. Scale bars = 100 µm (left panels), 50 µm (Insets). CA, cornu ammonis; DG, dentate gyrus; ERBB4, Erb-B2 receptor tyrosine kinase 4; GLP2R, glucagon-like peptide 2 receptor; KO, SREBP-1c knockout mice; NDN, necdin; PCL, pyramidal cell layer; WT, wild-type mice.

To determine if the protein expression pattern that we identified corresponded with the results of the qRT-PCR analyses, western blot analyses were performed for selected differentially expressed genes, *Glp2r*, *Ndn,* and *Erbb4* (Figure 4A). The results revealed that the total protein expression that was uncovered for decreased levels of GLP2R (M = 0.4345, SD = 0.1226; t(4) = 7.545, *p* = 0.0017) and NDN (M = 0.5140, SD = 0.08201; t(4) = 6.419, *p* = 0.0030), and increased level of ERBB4 (M = 1.935, SD = 0.3769; t(4) = 3.699, *p* = 0.0209) in the hippocampus was in agreement with the results of the RNA-seq analysis and qRT-PCR. Furthermore, these findings were validated by the immunohistochemical results that revealed similar alterations in the expression patterns of GLP2R (Figure 4B), NDN (Figure 4C), and ErBB4 (Figure 4D) in the hippocampus. In particular, GLP2R and NDN were strongly expressed in the PCL of the hippocampal CA3 subregion, which corresponds with the localizations of intense SREBP expressions found in this subregion. However, ERBB4 was expressed extensively in all subregions of the hippocampus.

Experimentally, we were able to validate our main results by performing various analyses that generated similar results, and therefore, in conclusion, based on the aforementioned findings, we can speculate that the aberrations in the behavioral patterns of the KO mice are potentially related to the differential gene expression of the seven genes that we identified. This is discussed in further detail in the section below.

## 3. Discussion

We initially reported on brain dysfunction and schizophrenia-like behaviors exhibited by SREBP-1c KO mice [15]. In this study, we considered that determining whether there were previously unidentified new genes involved in the brain functions of SREBP-1c KO mice would provide new evidence for the underlying mechanism behind these brain dysfunctions. We subjected the hippocampi of WT littermate and SREBP-1c KO mice to RNA-seq to assess the molecular variances. Consequently, this study uncovered and validated seven novel genes that might be related to SREBP-1c function in the hippocampus, and their expressions were confirmed by the results of biochemical assays.

As mentioned earlier, our group has previously reported that SREBP-1c KO mice exhibit schizophrenia-like behaviors accompanied by aberrations in the prefrontal cortex and the hippocampal gamma-aminobutyric acid signaling process [15]. As the brain tissue is mainly composed of lipids, we examined if the ablation of SREBP-1c, a major lipid homeostasis regulator that is expressed abundantly in the rodent brain, would also result in abnormalities in the expression of other genes that may be related to hippocampal dysfunction. First, we measured the levels of the two SREBP-1 isoforms, SREBP-1a and -1c, and of SREBP-2 in the hippocampus. We confirmed the complete knockdown of *Srebf1c* mRNA and ~43% decrease of SREBP-1 protein levels in the KO mice and found a slight but significant increase in the *Srebf1a* and *Srebf2* mRNA and SREBP-2 protein levels. After the confirmation of *Srebf1c* gene ablation in the KO mouse hippocampus, we performed genome-wide gene profiling in the hippocampus of the WT and SREBP-1c KO mice, and the presence of a total of seven DEGs (*Ndn*, *Erbb4*, *Srebf1*, *Il1r1*, *Glp2r*, *Gm16887*, and *Aox4*) was predicted. The results of the gene ontology analysis of the DEGs revealed that they were associated with signaling pathways (single organism signaling, cell surface receptor signaling pathway, transmembrane receptor protein tyrosine kinase signaling pathway, enzyme-linked receptor protein signaling pathway, and signal transduction), cell/neuron migrations (neuron migration, cell migration, and regulation of neuron migration), cellular responses (cellular response to organic substances and cellular response to chemical stimulus), transcription (positive regulation of transcription, DNA-templated transcription, positive regulation of nucleic acid-templated transcription, positive regulation of the RNA biosynthetic process, and transcription regulatory region DNA binding), cell motility, and the localization of cells. Thus, the gene ontology analysis results revealed enrichment of terms associated with signaling pathways, cellular responses, and transcription. As the *Srebf* gene codes for the transcription factors that regulate lipid homeostasis, we speculate that the DEGs identified in the SREBP-1c KO mouse model may be associated with the disturbances in the signaling pathways that are present in the hippocampus. Collectively, these aberrations in the signaling pathways could have led to the previously reported behavioral dysfunctions that were exhibited by the SREBP-1c KO mice [15].

GLP2R is highly expressed in the hippocampus [20] and is primarily involved in food intake regulation [21]. A proglucagon-derived peptide, glucagon-like peptide-2, serves as a neurotransmitter that is involved in the regulation of food intake, control of energy balance, and glucose homeostasis [21]. In this study, the hippocampal GLP2R mRNA/protein expression levels were found to be strikingly decreased in the SREBP-1c KO mice. Previous reports have implicated the involvement of GLP2R expression in the brain, and they have outlined that its decreased expression is related to the cognitive dysfunctions [22] owing to an impairment of neurogenesis. Additionally, there is a report outlining that GLP2R has a neuroprotective function against glutamate-induced excitotoxicity, as observed in cultured rat hippocampal cells [22]. Previously, we found that the SREBP-1c KO mice exhibited emotional dysregulations related to depression-like behavior (ex. increased immobility in a tail-suspension test) [15]. In a human brain study, GLP2R was significantly decreased in the dorsolateral prefrontal cortex, but not dysregulated within the hippocampus (just a decreasing trend of GLP2R) [23]. In contrast, decreased GLP2R resulted in cognitive impairment in Sprague-Dawley rats subjected to chronic cerebral hypoperfusion [22]. A decrease in GLP2R levels within the hippocampus of SREBP-1c KO mice may be related to the brain dysfunctions, even though the conflicting data between human and rodent studies should be considered for future studies on the relationship between schizophrenia and GLP2R.

NDN is a member of the MAGE (melanoma antigen) family [24], and a deficiency in this gene’s expression is primarily associated with the Prader–Willi syndrome (PWS) [25]. The PWS is a developmental and behavioral neurological disorder that is predominantly characterized by both incapacitating physical and developmental disabilities, including mental retardation and learning disabilities [26,27]. Neurologically, this gene has been reported to promote neuronal differentiation [28,29], cell survival [29], and axonal outgrowth [30]. In this study, we found significantly decreased NDN expression levels in the SREBP-1c KO mice, suggesting that SREBP-1c ablation is detrimental to the hippocampus as normal neuronal function related to differentiation, survival, and outgrowth may be subsequently impaired.

In addition, this study demonstrated that SREBP-1, GLP2R, and NDN expressions are observed more intensely in the CA3 subregion of the hippocampus in both WT and SREBP-1c KO mice, with the KO mice exhibiting a significantly decreased expression level for all related proteins. The CA3 subregion has been associated with memory formation and high susceptibility to seizures and neurodegeneration [31]. Thus, this finding suggests that a decrease in the expression levels of both GLP2R and NDN in the CA3 subregion may result in aberrations in the pivotal roles played by pyramidal neurons that are localized in this subregion. Our findings further support the association of impaired hippocampal GLP2R and NDN signaling with the behavioral aberrations observed in SREBP-1c KO mice. However, a PPI network analysis revealed that neither protein has a known physical nor functional interaction with SREBP-1. Thus, further studies are required to verify the co-relationship between lipid homeostasis, as regulated by SREBP-1, and these signals in the CA3 sub-region of the hippocampus.

IL1R1 is a vital mediator involved in various cytokine-induced immune and inflammatory processes [32], as such it is ubiquitously expressed in various organs throughout the body [33]. A PPI analysis in mice revealed that IL1R1 has both predicted and confirmed interactions with SREBPs, suggesting a direct relationship between the decrease in SREBP-1 and subsequent decrease in IL1R1 expression. In the central nervous system, IL1R1 expression bears predilection with the DG neurons of the hippocampus [34,35]. Variations in IL1R1 expression have been studied with a focus on traumatic [36,37,38] and ischemic brain disease models [39,40], mood disorders [41,42], and schizophrenia [43,44]. Some studies have demonstrated that the suppression or ablation of IL1R1 has a reparative role against neurotoxicity [45] and that it prevents the development of anxiety-like behavior in mice [46]. In contrast, another study found that the KO of IL-1R in mice exacerbated virus-induced cell death and neuroinflammation [47]. Because of the wide array of functions that IL1R1 is responsible for in different types of brain cells, further elucidation is required regarding the involvement and role of IL1R1 in the pathogenesis of brain disorders and their corresponding animal models. In our study, the decrease in IL1R1 expression observed in the hippocampus of the SREBP-1c KO mice may have either contributed to the development of the neurological dysfunctions or acted as a compensatory mechanism; which may or may not be related with the decrease in SREBP-1c. Thus, further studies are needed to elucidate this.

The neuregulin 1 receptor, ERBB4, is a member of the ERBB subfamily of type I receptor tyrosine kinases and modulates cell proliferation, growth, and differentiation [48,49,50,51]. Regarding the possible interaction of ERBB4 with SREBPs, a PPI network analysis revealed that ERBB4 has predicted interactions with SREBP-1. Moreover, this protein has also been implicated as an activator of SREBP-2 which results in an increase of low-density lipoprotein uptake and cholesterol biosynthesis, as well as SREBP-1 mediated fatty acid synthesis via the phosphoinositide 3-kinase signaling pathway in mammary epithelial cells [52]. In relation to schizophrenia, there are contrasting reports regarding the effects of ERBB4 expression levels. Del Pino et al. [53] demonstrated that the deletion of ERBB4 from fast-spiking interneurons might result in the development of schizophrenia-like behaviors while other groups have detected increased ERBB4 expression levels in the prefrontal cortex of schizophrenia patients [51,54]. Furthermore, the possible association of ERBB4 with schizophrenia may also be elucidated through the Wnt/β-catenin pathway, a well-established key player in the neurodevelopmental mechanisms involved in schizophrenia [55]; this pathway in conjunction with signaling cascades of schizophrenia-risk genes, DISC1 and NRG, has been reported to converge with ERBB4 [56]. We have previously reported that SREBP-1c KO mice exhibit schizophrenia-like behaviors, such as increased aggressiveness, decreased social interaction, and sensory-motor gating deficits, all of which are symptoms exhibited by human schizophrenia patients [15]. In accordance with the results of the latter research groups, we found significantly increased ERBB4 mRNA/protein expression levels in the hippocampus of the SREBP-1c KO mice in our study. The ERBB4 expression was found extensively in all subregions of the hippocampus without particular predilection to any area. This provides interesting evidence that supports the involvement of ERBB4 signaling in the hippocampus of SREBP-1c KO mice that exhibit schizophrenia-like behaviors; however, further studies are needed to confirm the specific role of ERBB4 signaling in hippocampal lipid homeostasis of SREBP-1c KO mice.

*Aox4* is one of the four complements of the aldehyde oxidase gene present in rodents [57]. The principal function of *Aox4* is its involvement in the breakdown of drugs and compounds in the liver; however, its exact physiological substrates and functions are still unknown [58]. In mice, the depletion of *Aox4* has led to disruptions in the expression of circadian-rhythm genes coupled with a decrease in locomotor activity [59]. Previously, we have reported on increased locomotor activity in SREBP-1c KO mice [15]. A significant increase in *Aox4* gene expression levels in the hippocampus of the SREBP-1c KO mice conceivably played a role in the subsequent hyperlocomotion exhibited by the KO mice. Therefore, this hypothesis may be supported by the findings of Terao et al. [59]; however, further studies are warranted to confirm this proposition.

Collectively, this study further provides evidence for the possible relationship between SREBP-1c and schizophrenia; it is the first study to reveal other molecules that are altered in conjunction with the depletion of SREBP-1c signaling in the mouse hippocampus.

## 4. Materials and Methods

### 4.1. Animals

In this study, we used 3–4-month-old SREBP-1c KO mice that were a generous gift from Dr. Timothy F. Osborne (Sanford Burnham Prebys Medical Discovery Institute, Orlando, FL, USA), and we maintained them on a C57BL/6J strain background using a previously described method [15]. The mice were housed in standard cages and were maintained on a 12:12 h light/dark cycle at room temperature (RT) of 25 °C in a specific pathogen-free facility. Food and water were provided ad libitum. All the experimental and animal handling procedures were performed in accordance with the guidelines of the institutional care and use committee of Chonnam National University (6 January 2017; CNU IACUC-YB-2017-01) and in line with the National Institute of Health’s (NIH) Guide for the Care and Use of Laboratory Animals. Every effort was made to minimize the number of animals used and the suffering they experienced.

### 4.2. RNA Isolation and RNA-seq

The hippocampi (n = 2 mice/group) were obtained from the mice following decapitation. Total RNA was isolated using an RNAeasy Lipid Tissue Mini Kit (Qiagen, Hilden, Germany) by following the manufacturer’s instructions. RNA quality and concentration were measured using the NanoDrop ND- 2000 system (Thermo Fisher Scientific, Waltham, MA, USA) and Bioanalyzer 6000 Nano Kit in the Bioanalyzer 2100 (Agilent, Santa Clara, CA, USA). Unpaired-end RNA-seq was performed on the RNA isolated from the hippocampi. The Quant-Seq 3’ Library Prep kit (Lexogen, Vienna, Austria) was used to obtain the sequencing libraries. The samples were sequenced via Illumina NextSeq500 (Illumina, San Diego, CA, USA). The reads with a length of less than 25 bp and a Phred quality score of less than 23 were trimmed using BBduk (version 38.44, Joint Genome Institute (JGI), Berkeley, CA, USA). The genome index was built using Star (version 2.7.3a) [60] along with Ensembl GRCm38 (version 98, EMBL-EBI, Cambridgeshire, UK).

### 4.3. DEGs, Enrichment Analysis and PPI Analysis

The trimmed reads were aligned to the indexed genome using the Star application. The aligned files were converted to a BAM file and were indexed to be analyzed further using Samtools (version 1.9, La Jolla, CA, USA). The SummarizedExperiment (version 1.12.0) [61]. Bioconductor R package was used to measure the read counts. The DEG analysis was conducted using the EdgeR (version 3.24.3) Bioconductor R package [18]. The genes with an FDR of less than 5% (FDR < 0.05) were considered differentially expressed. To analyze the gene ontology enrichment of the DEGs, the Database for Annotation, Visualization, and Integrated Discovery (DAVID, version 6.8) [62,63] was used.

The STRING database provides information that integrates both experimental and predicted physical or functional protein interactions [19]. To identify possible (existing or hypothetical) physical or functional interactions between the DEGs identified and SREBPs, a protein–protein interaction network was constructed by employing: text-mining, experiments, databases, co-expression and gene fusion analysis as sources of active interactions; using *Mus musculus* as the species of interest. A medium confidence of ≥ 0.400 was set for the minimum required interaction score, with nodes corresponding to the proteins and the edges representing protein–protein associations (Supplementary Figure S1).

### 4.4. RNA Extraction, cDNA Synthesis, and qRT-PCR

The hippocampi (n = 8 mice/group) were collected and digested in Trizol reagent for RNA extraction based on the manufacturer’s instructions (RNAeasy Lipid Tissue Mini Kit; Qiagen). Reverse transcription was performed using the Superscript™ II Reverse Transcriptase kit (Invitrogen, Carlsbad, CA, USA). The resulting complementary DNA (cDNA) was diluted with RNase-free water to achieve a final concentration of 8 ng/μL, and the samples were stored at −70 °C. The quantitative PCR was performed with TOPreal™ qPCR 2XPreMIX (TaqMan Probe) (Enzynomics, Daejon, Korea) using the LineGene 9600 Plus machine (BIOER, Hangzhou, China) according to the manufacturer’s instructions. The qRT-PCR primers are listed in Supplementary Table S3. The annealing temperature for the reaction was 58.5 °C, and the built-in software generated the amplification curves and computed the threshold cycle values. All the readouts were normalized using the β-actin reference gene. Via the 2^−ΔΔCT^ method [64], data were expressed as mean relative values compared to the values of the WT controls.

### 4.5. Western Blot

The western blot method was carried out similarly to a previous study [65]. In brief, the hippocampal samples (n = 3 mice/group) were sonicated for 10 s in buffer H (50 mM β-glycerophosphate, 1.5 mM ethylene glycol tetraacetic acid, 1 mM dithiothreitol, 10 μg/mL aprotinin, 1 mM phenylmethanesulfonyl fluoride, 0.1 mM sodium orthovanadate (Na_3_VO_4_), 10 μg/mL leupeptin, and 2 μg/mL pepstatin; pH 7.4) before adding the sodium dodecyl sulfate (SDS) sample buffer (4×) and heating for 10 min at 100 °C. The proteins were separated on 10% sodium dodecyl sulfate–polyacrylamide gel (SDS-PAGE) (Bio-Rad; Hercules, CA, USA) and were blotted onto polyvinylidene difluoride membranes (Amersham Hybond; GE Healthcare Life Sciences membranes; Pittsburgh, PA, USA). The membranes were blocked for 1 h with 1% (*v*/*v*) normal goat serum (NGS) and 0.5% (*v*/*v*) fetal bovine serum in phosphate-buffered saline (PBS) containing 0.1% (*v*/*v*) Tween 20 (PBS-T; pH 7.4) at RT and were subsequently incubated overnight with the following primary antibodies diluted with PBS-T: rabbit anti-SREBP-1 (1:1000; Abcam; Cambridge, UK), rabbit anti-SREBP-2 (1:1000; Invitrogen), rabbit anti- GLP2R (1:1000; Biorbyt; Cambridge, UK), rabbit anti-NDN (1:1000; Abcam), and mouse anti-ERBB4 (1:1000; Invitrogen). After thorough washing, the membranes were incubated with horseradish peroxidase-conjugated anti-rabbit or -mouse secondary antibody (1:10,000; Thermo Fisher Scientific; MA, USA) for 1 h before subsequent signals were detected using a chemiluminescence kit (SuperSignal West Pico or Femto; Thermo Fisher Scientific; MA, USA). The signals were normalized with mouse anti-β-actin primary antibody (1:5000; Sigma-Aldrich; MO, USA). Each band’s optical density (OD; per mm2) was calculated using the C-DiGit Blot Scanner 3600 (Li-Cor, Lincoln, NE, USA), and the ratio of each band’s density relative to that of β-actin was calculated using ImageJ (version 1.52p) [66]. A value of 1 was allocated to the mean control intensity, and the proportional shifts in the intensity levels of each sample were represented as the relative OD. The relative OD was averaged by a group and represented as mean ± standard error (SE).

### 4.6. Immunohistochemistry Examination of Free-Floating Sections

The immunohistochemistry protocol that was employed was based on a previous study [67]. Briefly, the mice (n = 3 mice/group) were perfused with 4% (*w*/*v*) paraformaldehyde (PFA; Sigma-Aldrich) in PBS, and subsequently, the brains were collected and immersed into 4% (*w*/*v*) PFA in PBS for 2–3 days. Brain cryoprotection was performed with 30% (*w*/*v*) sucrose for 4 days. Hippocampal brain sections were collected at approximately 2.12 mm posterior to the bregma. Following this, 30-μm-thick coronal slices were prepared using a sliding microtome (SM2010R; Leica Microsystems, Wetzlar, Germany), and they were stored in PBS at 4 °C. Endogenous peroxidase activity was blocked using 0.3% (*v*/*v*) hydrogen peroxide for 20 min. Further, 5% (*v*/*v*) NGS (Vector ABC Elite Kit; Vector Laboratories, Burlingame, CA, USA) in 0.3% (*v*/*v*) Triton X-100 was used to block non-specific reactions for 1 h. The sections were then incubated overnight at 4 °C in an antibody dilution buffer (Invitrogen) with the following primary antibodies: rabbit anti-SREBP-1 (1:200; Abcam), rabbit anti-SREBP-2 (1:200), rabbit anti-GLP2R (1:200; Biorbyt), rabbit anti-NDN (1:200; Abcam), and mouse anti-ERBB4 (1:500; Invitrogen). After thorough washing, the sections were subsequently incubated with biotinylated goat anti-rabbit or -mouse immunoglobulin G (IgG) (Vector ABC Elite Kit; Vector Laboratories) for 1 h at RT. Following this, the sections were incubated for 1 h at RT with an avidin–biotin peroxidase complex (Vector ABC Elite Kit; Vector Laboratories) according to the manufacturer’s instructions. After washing the sections, diaminobenzidine substrate (contained in the DAB kit; Vector Laboratories) was applied to them for the peroxidase reaction based on the manufacturer’s instructions. In a few test sections, primary antibodies were omitted in each experiment.

### 4.7. Statistical Analysis

The data are expressed in terms of means ± SD. The normality of data were determined by the Shapiro–Wilk test. The results were evaluated to identify variances between the WT and SREBP1c KO groups using independent two-tailed Student t-tests that were conducted using the GraphPad (version 8.4, GraphPad Software, San Diego, CA, USA) and R studio libraries (R Studio, Boston, MA, USA), ggplot2, and ggsignif. A *p*-value of less than 0.05 was considered to be statistically significant in all the analyses. Power analysis to determine the minimum number in a sample size required per group was performed using “La Morte’s Power Calculator” at alpha level 0.05 to achieve a power of at least 90% [68]. The minimum required sample sizes obtained using this method were: at least n = 5 mice for mRNA relative expression analysis; n = 3 mice for relative optical density values obtained from western blot analysis. However, due to costs and technical limitations, the sample size utilized for the RNA-seq analysis was limited to 2 biological replicates, in accordance with previous studies [69,70]. Possible decreases in the true positive rate were compensated by the use of the EdgeR tool [71].

## 5. Conclusions

In summary, our study highlights the presence of alterations in the expression of novel gene candidates (closely related to schizophrenia-like behavior) in the hippocampus of SREBP-1c KO mice. Based on our results, we propose that the findings in the schizophrenia-like behavioral phenotype of SREBP1-c KO mice are closely linked to the differential gene expression of *Glp2r*, *Ndn*, *Il1r1*, *Erbb4*, and **Aox4**, which possibly results in changes in signaling transduction, cell differentiation, and cell survival within the hippocampal cell population. However, further scrutiny of the translational value from rodent models to human patients needs to be done to fully establish the relationship with the differentially expressed genes found in this study and the conjectured hippocampal functional alterations. Furthermore, due to the multifaceted hypotheses surrounding the etiology of schizophrenia, it would be worthwhile to explore the developmental transcriptome system of this model in future studies. This would provide an avenue for the elucidation of possible DEGs related to the neurodevelopmental hypothesis of schizophrenia’s etiology. Nevertheless, acquiring this information represents an important starting point for the establishment of new models for schizophrenia and provides a novel avenue for future studies that may explore new therapeutic targets to treat schizophrenia.

## Supplementary Materials

Supplementary materials can be found at https://www.mdpi.com/1422-0067/21/11/4131/s1. Supplementary Table S1: Result of EdgeR analysis in the hippocampus of SREBP-1c knockout mice. Supplementary Table S2: Summary of gene ontology analysis in the hippocampus of SREBP-1c knockout mice. Supplementary Table S3: Primer sequences used for qRT-PCR analysis. Supplementary Figure S1: STRING protein–protein interaction (PPI) analysis of selected DEGs and SREBP genes.

## Abbreviations

SREBPs	Sterol regulatory element-binding proteins
*Srebf*	Sterol regulatory element-binding transcription factor
FA	Fatty acids
DNA	Deoxyribose nucleic acid
KO	Knockout
WT	Wild-type
DEGs	Differentially expressed genes
PPI	Protein-protein interaction
STRING	Search Tool for the Retrieval of Interacting Genes database
qRT-PCR	Quantitative real-time polymerase chain reaction
mRNA	Messenger ribonucleic acid
RNA-seq	RNA-sequencing
CA	Cornu ammonis
DG	Dentate gyrus
PCL	Pyramidal cell layer
FDR	False discovery rate
*Glp2r*	Glucagon-like peptide 2 receptor
*Ndn*	Necdin
*Il1r1*	Interleukin 1 receptor type I
*Erbb4*	Erb-B2 receptor tyrosine kinase 4
*Aox4*	Aldehyde oxidase 4
MAGE	Melanoma antigen
PWS	Prader–Willi syndrome
SDS-PAGE	Sodium dodecyl sulfate–polyacrylamide gel
NGS	Normal goat serum
PBS	Phosphate-buffered saline
PBS-T	Phosphate-buffered saline + Tween 20
RT	Room temperature
OD	Optical density
SE	Standard error
PFA	Paraformaldehyde
