# Supplementary material for: Transcriptome Profiling Reveals Novel Candidate Genes Related to Hippocampal Dysfunction in SREBP-1c Knockout Mice
Source: Int J Mol Sci. 2020 Jun 10;21(11):4131. doi: 10.3390/ijms21114131 (PMC7313053; doi:10.3390/ijms21114131)
Supplement: Supplementary file 1 [file ijms-21-04131-s001.zip › Supplementary_Figure 1_Ang et al.pdf]

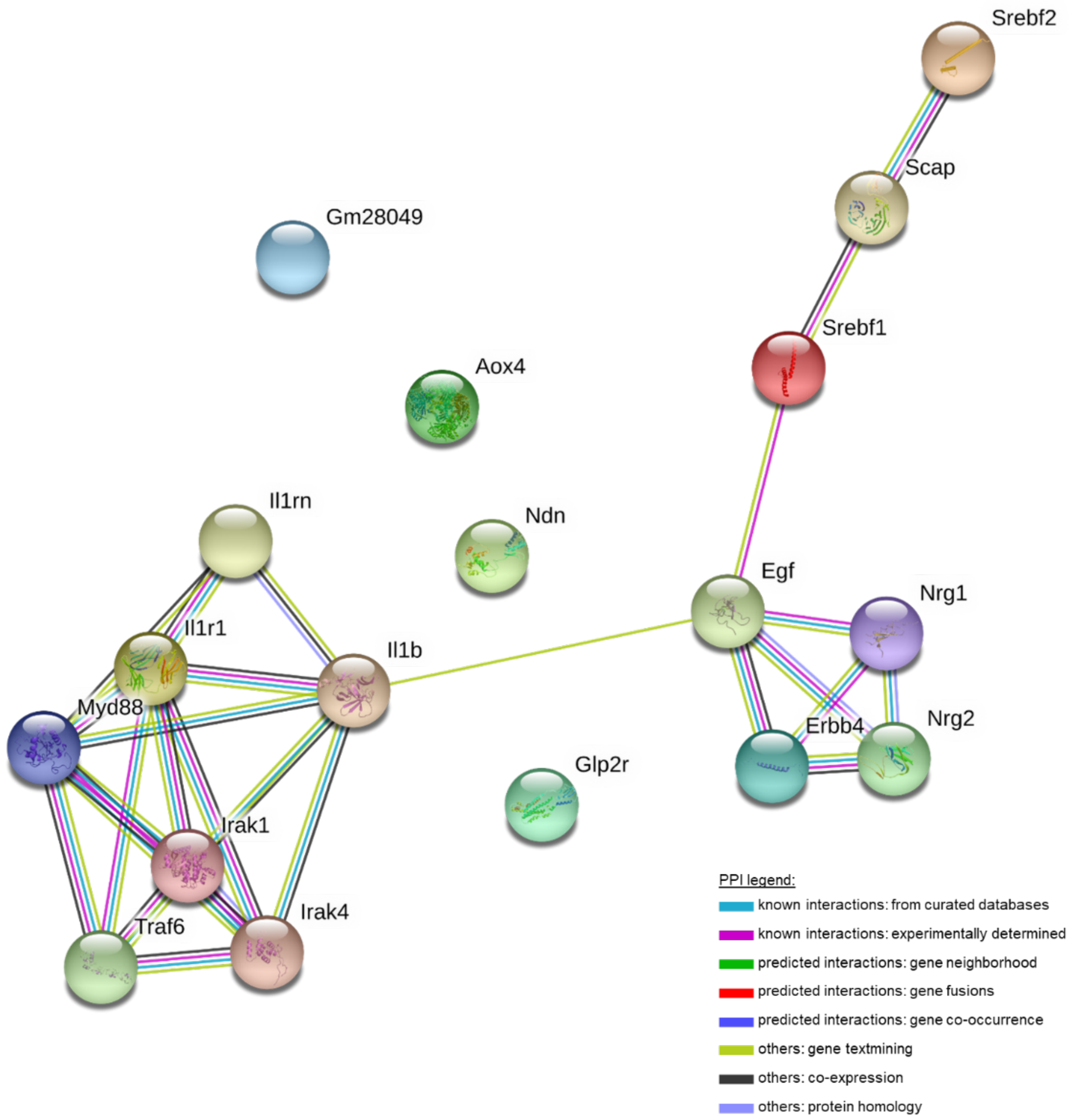

Supplementary Figure 1. STRING protein-protein interaction (PPI) analysis of selected DEGs and SREBP genes. PPI interaction network was constructed using the STRING database (https://string-db.org). Sources of active interactions was set to: text-mining, experiments, databases, co-expression and gene fusion. Species of interest was set to '*Mus musculus*'. Minimum required interaction score was set to ≥ 0.400. The network contains 18 nodes with 28 edges (*vs*. 14 expected edges); clustering coefficient 0.582; enrichment p-value .000451; average node degree 3.11. PPI legends indicate the type of interaction evidence. Aox4, Aldehyde oxidase 4; DEG, Differentially expressed genes; Egf, Pro-epidermal growth factor; Erbb, Receptor tyrosine-protein kinase erbB-4; Glp2r, Glucagon-like peptide 2 receptor; Gm28049, Predicted gene, 16867; Il1b, Interleukin-1 beta; Il1r1, Interleukin-1 receptor type 1; Il1rn, Interleukin-1 receptor antagonist protein; Irak1, Interleukin-1 receptor-associated kinase 1; Irak4, Interleukin-1 receptor-associated kinase 4; Myd88, Myeloid differentiation primary response protein MyD88; Ndn, Necdin; Nrg1, Neuregulin-1 type III beta1-a; Nrg2, Pro-neuregulin-2; Scap, Sterol regulatory element-binding protein cleavage-activating protein; Srebf1, Sterol regulatory element-binding protein 1; Srebf2, Sterol regulatory element-binding protein 2; Traf6, TNF receptor-associated factor 6.
