# Supplementary material for: Transcriptome Profiling Reveals Novel Candidate Genes Related to Hippocampal Dysfunction in SREBP-1c Knockout Mice
Source: Int J Mol Sci. 2020 Jun 10;21(11):4131. doi: 10.3390/ijms21114131 (PMC7313053; doi:10.3390/ijms21114131)
Supplement: Supplementary file 1 [file ijms-21-04131-s001.zip › Ang et al._Supplementary Table 3.docx]

Supplementary Table 3. Primer sequences used for qRT-PCR analysis.

| Gene | NCBI Accession No. | Primer sequence | | product size |
| --- | --- | --- | --- | --- |
| *Srebf1c* | NM_001358314.1 | FWD | 5'-ATCGGCGCGGAAGCTGTCGGGGTAGCGTC-3' | 116 |
|  |  | RVS | 5'-ACTGTCTTGGTTGTTGATGAGCTGGAGCAT-3' |  |
| *Srebf1a* | NM_011480.4 | FWD | 5'-TAGTCCGAAGCCGGGTGGGCGCCGGCGCCAT-3' | 106 |
|  |  | RVS | 5'-GATGTCGTTCAAAACCGCTGTGTGTCCAGTTC-3' |  |
| *Srebf2* | NM_033218.1 | FWD | 5′-CACAATATCATTGAAAAGCGCTACCGGTCC-3′ | 200 |
|  |  | RVS | 5′-TTTTTCTGATTGGCCAGCTTCAGCACCATG-3′ |  |
| *Glp2r* | BC044746.2 | FWD | 5'-GTGGGCTACTCCCTGTCTCT-3' | 121 |
|  |  | RVS | 5'-TCAGGATGAACGAAGCGAACA-3' |  |
| *Ndn* | NM_010882.3 | FWD | 5'-CGTGTTGGTGAAGGACCAGA-3' | 129 |
|  |  | RVS | 5'-AACACTCTGGCGAGGATGAC-3' |  |
| *Il1r1* | XM_006495714.3 | FWD | 5'-AGAAGGTCTTAGCTGGTGCG-3' | 119 |
|  |  | RVS | 5'-CTCGGGGGTCACATTCCTTC-3' |  |
| *Gm16867* | XM_017316303.1 | FWD | 5'-GGCCTCTTCTTCTCCAGCTAT-3' | 104 |
|  |  | RVS | 5'-CTTAAAGTGGGAACCTCCTCC-3' |  |
| *Erbb4* | XM_006495692.3 | FWD | 5'-TCTTTATGGCCCCACTCAGG-3' | 166 |
|  |  | RVS | 5'-GACCAACAGCTTTCGAGCCT-3' |  |
| *Aox4* | XM_006496287.2 | FWD | 5'-CCTGAGGACCGACATCTTCG-3' | 139 |
|  |  | RVS | 5'-GAGCACACCTTTTGGGGAGT-3' |  |

Abbreviations: *Aox4* , aldehyde oxidase 4; *Erbb*, Erb-B2 Receptor Tyrosine Kinase 4; *Glp2r*, Glucagon-like peptide 2 receptor; *Il1r1*, Interleukin 1 receptor, type I; *Ndn*, Necdin; *Srebf*, sterol regulatory element-binding transcription factor; FWD, forward primer; RVS, reverse primer.
